# Remediation Capacity of Different Microalgae in Effluents Derived from the Cigarette Butt Cleaning Process

**DOI:** 10.3390/plants11131770

**Published:** 2022-07-03

**Authors:** Carolina Chiellini, Lorenzo Mariotti, Thais Huarancca Reyes, Eduardo José de Arruda, Gustavo Graciano Fonseca, Lorenzo Guglielminetti

**Affiliations:** 1 Department of Agriculture, Food and Environment, University of Pisa, 56124 Pisa, Italy; carolina.chiellini@ibba.cnr.it (C.C.); lorenzo.mariotti@unipi.it (L.M.); lorenzo.guglielminetti@unipi.it (L.G.); 2 Institute of Agricultural Biology and Biotechnology, Italian National Research Council, 56124 Pisa, Italy; 3 Centro di Ricerche Agro-Ambientali “E. Avanzi”, University of Pisa, 56122 Pisa, Italy; 4 Faculty of Exact Sciences and Technology, Federal University of Grande Dourados, Dourados 79804-970, MS, Brazil; eduardoarruda@ufgd.edu.br; 5 Faculty of Natural Resource Sciences, School of Business and Science, University of Akureyri, 600 Akureyri, Iceland; gustavo@unak.is

**Keywords:** anthropogenic litter, wastewater, bioremediation, microalgal strains, photosynthetic pigments

## Abstract

Microalgal-based remediation is an ecofriendly and cost-effective system for wastewater treatment. This study evaluated the capacity of microalgae in the remediation of wastewater from cleaning process of smoked cigarette butts (CB). At laboratory scale, six strains (one from the family Scenedesmaceae, two *Chlamydomonas* *debaryana* and three *Chlorella* *sorokiniana*) were exposed to different CB wastewater dilutions to identify toxicity levels reflected in the alteration of microalgal physiological status and to determine the optimal conditions for an effective removal of contaminants. CB wastewater could impact on microalgal chlorophyll and carotenoid production in a concentration-dependent manner. Moreover, the resistance and remediation capacity did not only depend on the microalgal strain, but also on the chemical characteristics of the organic pollutants. In detail, nicotine was the most resistant pollutant to removal by the microalgae tested and its low removal correlated with the inhibition of photosynthetic pigments affecting microalgal growth. Concerning the optimal conditions for an effective bioremediation, this study demonstrated that the *Chlamydomonas* strain named F2 showed the best removal capacity to organic pollutants at 5% CB wastewater (corresponding to 25 butts L^−1^ or 5 g CB L^−1^) maintaining its growth and photosynthetic pigments at control levels.

## 1. Introduction

Cigarette butts (CB) are the most littered item in the world, which are usually found spread everywhere from urban areas to even protected areas [1]. CB contain a variety of toxic compounds accumulated during smoking such as benzene, polycyclic aromatic hydrocarbons, pyridine and heavy metals, which can leach into the environment and affect all ecosystems [2]. Moreover, practical operational aspects are lacking at the regulatory level as the current disposal systems for CB are landfilling and incineration, which are unsustainable and release hazardous contaminants to the environment [3,4]. Therefore, alternative solutions to tackle this waste are urgently needed. Recently, Mariotti et al. [5] proposed a novel solution to recycle filters of CB into a soilless substrate for growing ornamental plants in urban spaces. However, the CB cleaning process used in Mariotti et al. [5] resulted in a contaminated wastewater, which must be treated before its reuse or release to the environment.

Algae comprise a large and heterogeneous group of mostly photosynthetic organisms, which are the primary producers of food chains in the ecosystems and contribute about 40% of global photosynthesis [6]. Microalgae are single-celled microorganisms that occupy a dominant position in global ecosystems due to their nutritional simplicity, efficient dispersivity, and broad ecological amplitude [6]. Moreover, the capacity to use sunlight to fix carbon via photosynthesis is usually more efficient in microalgae than terrestrial crops, resulting in a high biomass generation [7]. Consequently, the accumulation of carbohydrates, oil, sugar, proteins, cellulose, polymers and bioactive compounds in microalgae can be used as biofuel, feed and to produce bioplastic materials [8]. Moreover, many microalgae species have the capacity to remove inorganic contaminants including phosphates, nitrates, ammonia, sulphates, calcium, sodium and heavy metals, as well as to degrade organic pollutants such as hydrocarbons, pharmaceuticals and even herbicides [9]. Accordingly, microalgae are considered important tools to improve the environmental impacts of the currently used wastewater treatment methods, resulting in multiple benefits such as nutrient recovery, biomass production, and water reutilization or discharge to the environment without adverse ecological impacts [10].

Therefore, the objective of this study was to assess the removal of pollutants in CB wastewater by microalgal-based remediation techniques. Since the isolation and selection of suitable microalgae are essential for efficient wastewater treatment, in the present study six natural isolates were screened. All microalgal strains were cultivated in different dilutions of CB wastewater, and their tolerance towards pollutants and the capacity of wastewater remediation were evaluated. This included the measurement of the production of photosynthetic pigments to evaluate the effect of pollutants on the physiological activity of microalgae, and the evaluation of the profile of wastewater pollutants at the end of the microalgal remediation process. This study will therefore provide the scientific evidence to treat the wastewater from CB cleaning process by microalgal remediation and reveal the potential value of some microalgal strains for further studies on a larger scale.

## 2. Materials and Methods

### 2.1. CB Collection and Cleaning Process

The CB collection, cleaning process and chemical characterization were as previously described [5]. Briefly, CB were collected (5 kg approximately) from public collectors in 10 different coffee bars located in the surroundings of the municipality of Capannori (Lucca, Italy). The cleaning process was performed in quadruplicate by an exhaust boiling of CB (100 g) in distilled water (1 L) for 10 min. The individual wastewaters were collected for their further treatment with different microalgae.

### 2.2. Microalgal Strains and Growth Conditions

Six microalgal strains were used in this work (Table 1). Five of these strains were previously isolated and characterized [11], namely F1 (from the family Scenedesmaceae), F2 and F3 (both related to *Chlamydomonas debaryana* Goroschankin species), F4 and R1 (both related to *Chlorella sorokiniana* Shihira and R.W. Krauss species), and are currently part of the collection of the Institute of Agricultural Biology and Biotechnology of the Italian National Research Council located in Pisa. The sixth microalga, strain “LG1”, was isolated from recycled CB substrate and then characterized as described below.

The microalgal strain LG1 was isolated from the surface of a recycled CB filter substrate used in the preliminary experiments of a previous study [5]. This substrate was collected in a petri dish and used to make an enrichment with the TAP medium, as described by Chiellini et al. [9]. Briefly, 1 cm^3^ of the substrate was cut with a sterile scalpel under biological flow, and put in a sterile flask with 50 mL sterile TAP medium [12]. After two weeks’ enrichment, the solution was greenish. The solution was diluted in sterile TAP medium (1:20 *v*/*v*), and a second enrichment was performed for two more weeks. Light microscope observation (Carl Zeiss Axioskop 20 EL-Einsatz 451487) allowed a dominant microalgal coccoid morphology to be recognized. Three 100 μL aliquots of the enrichment were streaked on TAP agar plates. This process was further repeated until a single morphology indicating the presence of a single strain was isolated. A single colony was picked up from the monoclonal microalgal culture in the petri dish, and pre-inoculated in a liquid TAP medium (50 mL) until a dense pre-culture (200 mL) was obtained. The strain was named “LG1”. All the microalgal strains were grown and maintained in growth chamber under controlled temperature (24/22 °C), and under a 16/08 h day-night cycle with PPFD of 70 μmol photons m^−1^ s^−1^.

### 2.3. Characterization of LG1 Strain and Phylogenetic Analysis

One mL of the monoclonal culture of strain LG1 was used for DNA extraction as described by Chiellini et al. [9]. The 18S rRNA gene was amplified as previously described [9] using a MultiGene OptiMax Thermal Cycler (Labnet, NJ, USA), and visualized by electrophoresis on 1% agarose gel; amplicons were purified by ethanol/EDTA/Na-acetate precipitation and sent to the sequencing service (BMR Genomics, Padova, Italy). The obtained sequences (forward and reverse) were analyzed and used to obtain a complete 18S rRNA gene sequence using the free software Chromas (http://technelysium.com.au/wp/chromas/; accessed on 17 November 2021). The NCBI Blast tool [13] allowed the determination of the preliminary affiliation of the newly isolated microalgal strain by comparing the sequence with all the sequences present in the international databases. A total of 41 sequences were selected for the phylogenetic analysis, comprehending the sequence of our new strain, and 40 high quality sequences selected in NCBI database, following the similarity criterion. Among the 40 selected sequences, ten were chosen as the outgroup, and were taxonomically related to *Chlamydomonas* spp. and *Dunaliella* spp. The 41 sequences were aligned with the BioEdit Software [14]; a Maximum Likelihood phylogenetic tree was constructed with the MEGA5 Software [15]; the robustness of the inferred trees was evaluated by 500 bootstrap resampling; the parameters chosen for the phylogeny were: Model/Method = General Time Reversible model; Rates among sites = Gamma distributed with invariant sites (G + I); Gaps = Use all sites; ML heuristic method = Nearest Neighbor Interchange (NNI); Branch swap filter = Strong.

### 2.4. Evaluation of Microalgal Strains in Remediation

The wastewater was filter-sterilized by a 0.45 µm cellulose acetate filter (Sartorius, Göttingen, Germany), and different wastewater dilutions in TAP medium were tested in quadruplicates as follows: 0 (herein after Control), 1, 2, 5, 10 and 25% (*v*/*v*). In 24-well plates (1.5 cm diameter, Greiner Bio-one, Kremsmünster, Austria) 200 µL of microalgae culture was added to 1800 µL of fresh TAP medium containing a series of wastewater dilutions. The remediation capacity of each microalgal strain was performed under the same growth conditions: 24/22 °C, 16/08 h day-night cycle and 70 μmol photons m^−1^ s^−1^ PPFD. An additional 24-well plate containing only wastewater dilutions in TAP medium (2000 µL) was included to evaluate the effect of growth conditions on the wastewater chemical composition, herein termed untreated wastewater (UWW). After 7 days, the cultures in each well were centrifuged at 3000× *g* for 10 min, and the supernatant and the microalgae pellet were collected separately for further analysis.

### 2.5. Analytical Determinations

Supernatants were dried under vacuum and diluted with acetone and heptane 50% (*v*/*v*). Analytes in the wastewater samples were determined by high-resolution GC-MS analysis, using a Saturn 2200 quadrupole ion trap mass spectrometer coupled to a CP-3800 gas chromatograph (Varian Analytical Instruments, Walnut Creek, CA, USA) equipped with a MEGA-SE54 HT capillary column (10 m; 0.15 mm i.d., 0.10 µm film thickness, MEGA s.n.c., Milan, Italy), as reported by Mariotti et al. [5]. Data acquisition was from 10 to 550 Da at a speed of 1.4 scan s^−1^. The identification of chromatogram peaks was conducted by comparing their mass spectra with the NIST library database. Quantification was performed using the relative abundance of the chromatogram peaks (instrument detection limit < 400 counts).

### 2.6. Photosynthetic Pigments of Microalgal Strains

In order to assess the health status of microalgal strains, photosynthetic pigments were determined in four biological replicates. Photosynthetic pigments, including chlorophyll *a* (Chl*a*), chlorophyll *b* (Chl*b*) and total carotenoids (Car), were extracted from microalgae pellets and analyzed as previously reported [16].

### 2.7. Statistical Analyses

Values presented are the means of four replicates. The Tukey’s test was used to determine the significant differences among means (*p* < 0.05), in which the statistical analysis was performed by STATISTICA for Windows version 14.0 (Stat-Soft, Inc., Tulsa, OK, USA) using a one-way analysis of variance.

To identify the relationships among the remediation capacity of microalgal strains at different concentrations of CB wastewater, based on physiological and analytical data, multiple factor analysis (MFA) was carried out [17]. The MFA was performed with the R software [18], using the packages “FactoMineR” and “factoextra” for the analysis and data visualization, respectively. The final plot in the picture was obtained in R software with the packages “ggpubr”, “ggsci” and “patchwork”. Data were normalized with Z-score calculation.

## 3. Results

### 3.1. Identification of the LG1 Strain

**Figure 1:**
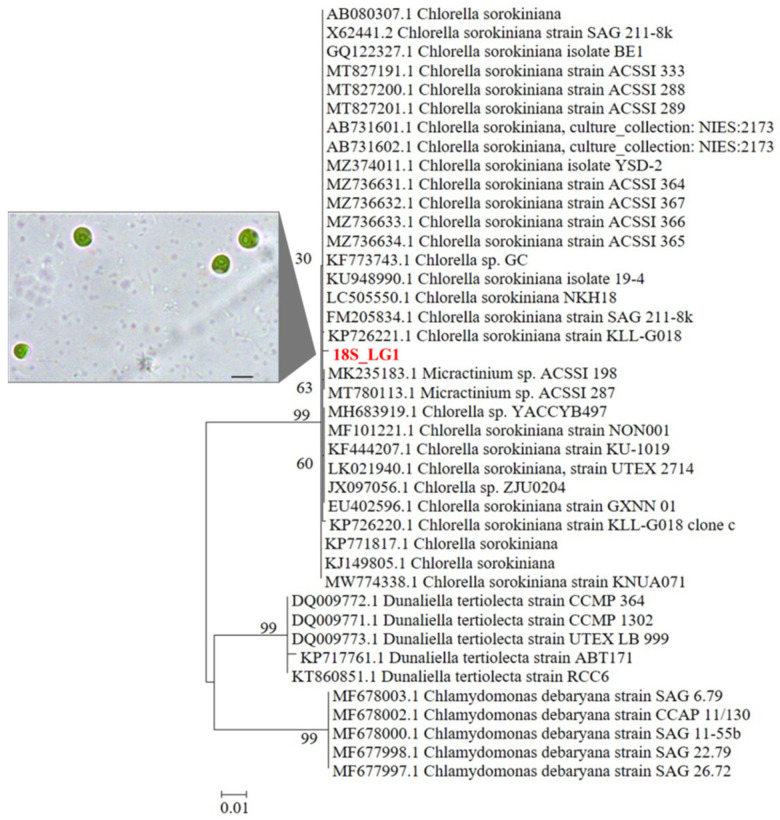
Phylogenetic tree reconstruction obtained with the Maximum Likelihood method on a total of 41 high quality sequences selected from the most similar to the sequences obtained for the LG1 strain. Inset: optical microscope image of LG1 cells (scale bar: 5 μm).

According to the phylogenetic analysis, the LG1 strain was taxonomically related to the *Chlorella sorokiniana* species (Figure 1).

### 3.2. Photosynthetic Pigments of Microalgal Strains

**Figure 2:**
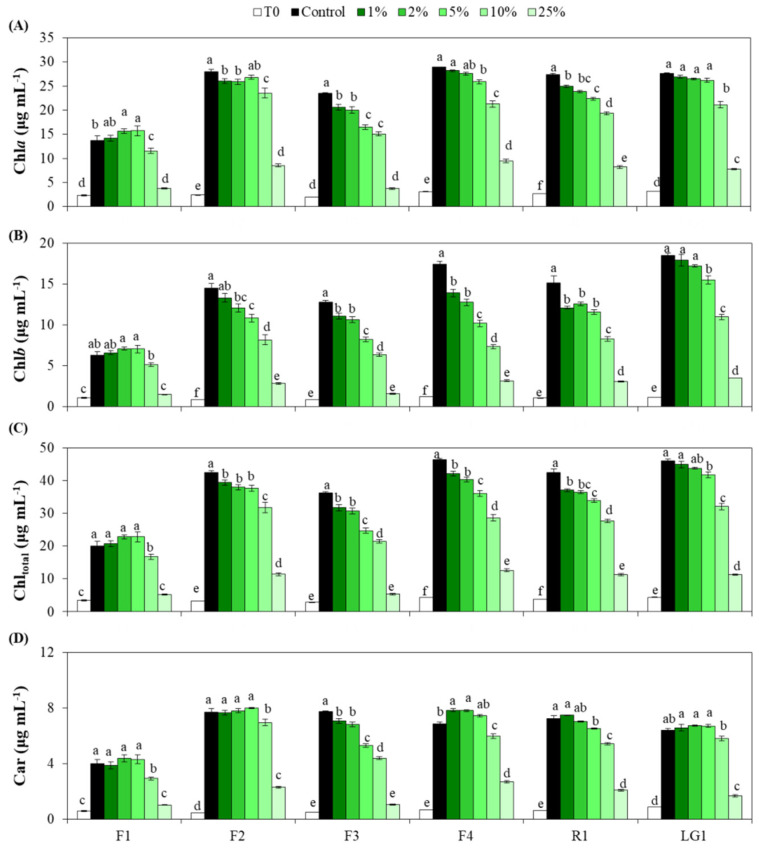
Effect of wastewater from cigarette butts (CB) cleaning process on photosynthetic pigments of six microalgal strains. (**A**) Chlorophyll *a* (Chl*a*), (**B**) chlorophyll *b* (Chl*b*), (**C**) total chlorophyll (Chl_total_) and (**D**) carotenoids (Car) were determined in each microalgal strain (F1, F2, F3, F4, R1 and LG1) at the beginning of the experiment (T0) and 7 days after treatment. Microalgal treatment included exposure to growth medium without CB wastewater (Control) or containing different CB wastewater dilutions (1, 2, 5, 10 and 25%). Different letters represent significant differences (*p* < 0.05) between treatments within the same strain. Data are expressed as means of 4 different replicates ± standard error (SE).

All microalgal strains showed a steep increase in chlorophyll *a* (Chl*a*), chlorophyll *b* (Chl*b*), total chlorophyll (Chl_total_) and carotenoids (Car) from the beginning of the experiment (T0) to 7 d under control conditions (TAP medium without CB wastewater) (Figure 2A–D). Chl*a* in F1 gradually increased with the wastewater concentration reaching the highest level with 5% wastewater; however, a significant and subsequent sharp decline was observed when the wastewater increased to 10 and 25%, respectively (Figure 2A). F3, F4 and R1 showed a gradual decrease in Chl*a* when the wastewater concentration increased to 10%, followed by an abrupt drop with 25% wastewater (Figure 2A). Differently, F2 and LG1 generally maintained Chl*a* at control levels when the wastewater concentration increased to 5%, followed by a decrease with 10 and 25% wastewater similar to the pattern of Chl*a* in F1 (Figure 2A). In general, Chl*b* and Chl_total_ in F1, F3 and R1 showed similar patterns to that of Chl*a* (Figure 2A–C). F2 and F4 showed a steep decline in Chl*b* with the increase in wastewater concentration, whereas the negative effect of wastewater on Chl*b* in LG1 was observed when exposed to more than 2% wastewater (Figure 2B). Chl_total_ in F2 generally exhibited similar dynamics to Chl*a* when contamination increased in the medium (Figure 2A,C), while Chl_total_ in F4 and LG1 showed similar trend to Chl*b* with the increase in wastewater concentration (Figure 2B,C). F1, F2 and LG1 maintained their stable levels of Car when the wastewater concentration increased to 5%, followed by a significant and subsequent sharp decline when contamination increased in the medium with the exception of LG1, which showed significant differences only at 25% wastewater with respect to the control (Figure 2D). Car in F3 showed similar patterns to that of Chl_total_ (Figure 2C,D). F4 showed a transient increase in Car when the wastewater increased to 2%, followed by a gradual and significant decrease with a higher wastewater concentration (Figure 2D). In contrast, Car in R1 started to show a progressive decline when CB wastewater was increased beyond 5% (Figure 2D).

### 3.3. CB Wastewater Subjected to Microalgal-Based Remediation

**Figure 3:**
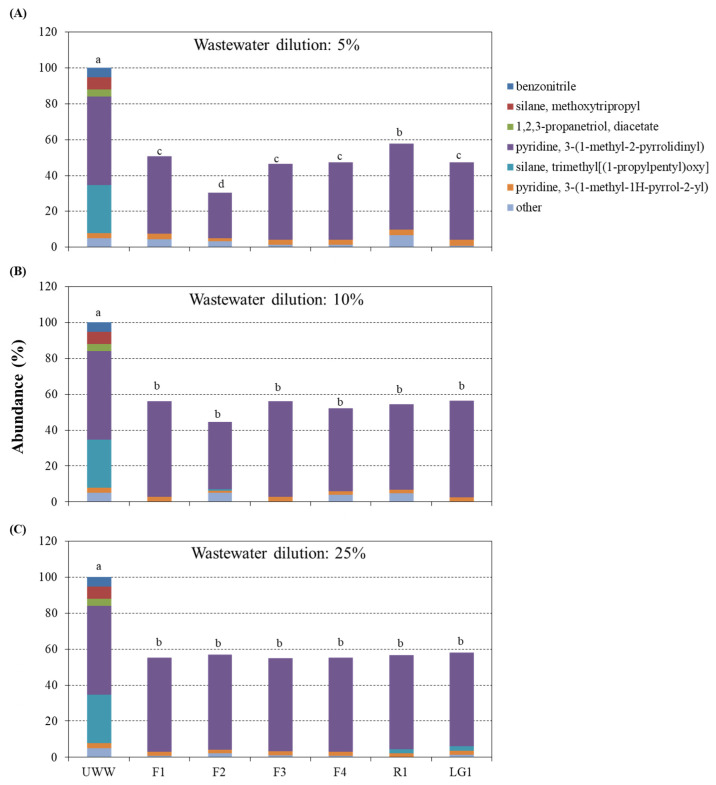
Chemical composition of the wastewater from the cigarette butts (CB) cleaning process subjected to microalgal-based remediation. Six microalgal strains (F1, F2, F3, F4, R1 and LG1) were exposed to different CB wastewater dilutions: (**A**) 5, (**B**) 10 and (**C**) 25%. The remediation capacity of each strain was evaluated after 7 days. UWW represents the respective CB wastewater dilution without microalgae under the same growth conditions for 7 days, for more details see Material and Methods. The total abundance of chemical compounds in UWW was expressed as 100%. The abundance of remaining compounds in wastewater after microalgal-based remediation was obtained by its comparison with UWW. Different letters represent significant differences (*p* < 0.05) between the total abundance of chemical compounds in UWW and microalgal treated wastewater. Data are the means of 4 different replicates.

In general, all microalgal strains showed a good ability to remediate CB wastewater and nicotine [pyridine, 3-(1-methyl-2-pyrrolidinyl)] was the most difficult compound to remediate among pollutants (Figure 3). In 5% wastewater, F2 showed the best capacity for removing pollutants compared with other strains (−69% with respect to UWW) followed by F3, F4, LG1 and F1 (−52%), and R1 (−42%) (Figure 3A). In contrast, no significant differences between the strains were observed when the wastewater concentration increased to 10 and 25% (Figure 3B,C). Thus, strains in 10% wastewater could remove on average 47% of pollutants with respect to UWW (Figure 3B), while those in 25% wastewater removed 44% of pollutants (Figure 3C).

### 3.4. Multiple Factor Analysis

**Figure 4:**
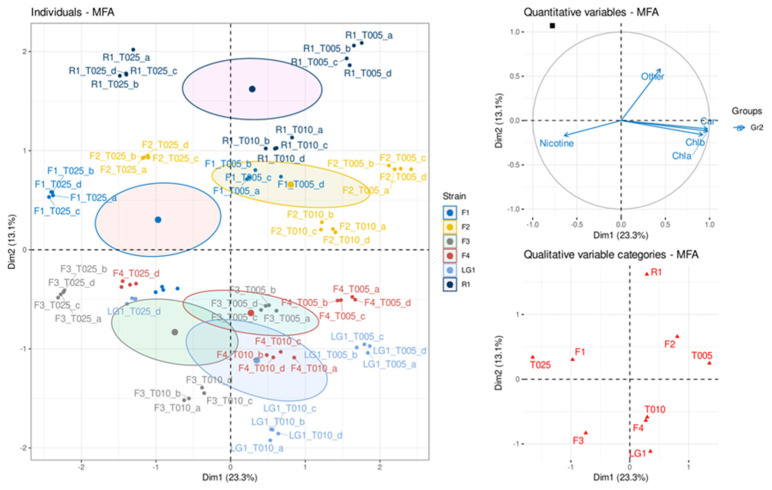
Multiple factor analysis (MFA) of physiological and analytical data in microalgal-based remediation of wastewater from cigarette butts (CB) cleaning process. T005: 5% CB wastewater dilution; T010: 10% CB wastewater dilution; T025: 25% CB wastewater dilution; a, b, c and d: indicate the replicates; Other: pollutants in CB wastewater other than nicotine.

The multiple factor analysis (MFA; Figure 4) revealed for each microalgal strain a distinct separation in three groups in relation to the CB wastewater concentration. Accordingly, the four replicates were exposed to the same CB concentration group together. According to the quantitative variables (Figure 4), strains F1, F3, F4 and LG1 exposed to 25% CB concentration, as well as F1 at 10% CB, were those showing the highest % of nicotine and the lowest amount of photosynthetic pigments. On the other side, strains F2 (5 and 10% CB), F1 (5% CB) and R1 (10% CB) were the strains showing the lowest nicotine concentration in the wastewater, as well as the highest amount of photosynthetic pigment content. An opposite behavior could be observed concerning other contaminants that were not nicotine (Figure 4). In this case, the MFA highlighted that the highest values (i.e., the lowest removal ability) were characterizing strains R1 and F2 (5% and 10% CB), and F1 (5% CB). On the contrary, strains F3 (all CB concentrations), F1 (10% CB) and LG1 (25% CB) seemed to remove the highest amount of other contaminants from the wastewater. According to the qualitative variables categories (Figure 4), the six strains were separated in two groups along the y axis; one group was comprised of strains F1, F2 and R1, and the other group strains F3, F4 and LG1. These two groups were related, respectively, to the content of “other” contaminants and to the content of nicotine in the wastewater.

## 4. Discussion

In this study, considering that native microalgal strains exhibit a better tolerance to diverse pollutants than commercial species [9], six strains resilient to particular environmental stress factors were screened for the remediation of organic pollutants in wastewater derived from the smoked CB cleaning process. For this purpose, different dilutions of CB wastewater for microalgal-based treatment were evaluated to identify the toxicity levels reflected in the alteration of microalgal physiological status and to determine the optimal conditions for the effective removal of contaminants.

Previous studies found a direct relationship between algal growth and Chl*a* content [19,20,21]. Here, results of Chl*a* indicate that microalgae growth was generally affected with a CB concentration of more than 2%. In detail, the cell growth of F3, F4 and R1 were inhibited at CB concentrations ≥ 5%, while that of F1, F2 and LG1 at CB ≥ 10%, suggesting that the latter had a better ability to resist or tolerate the toxicity of CB wastewater pollutants. Among pollutants, benzonitrile (UWW abundance: 5.2%); 1,2,3-propanetriol, diacetate (UWW abundance: 4.0%); and the silicon (Si)-based compounds such as silane, methoxytripropyl (UWW abundance: 6.8%) and silane, trimethyl [(1-propylpentyl)oxy] (UWW abundance: 26.7%) were completely or almost completely removed after microalgal-based treatment. Benzonitrile is an ingredient used in photosynthesis-inhibiting herbicides, which have differential effects depending on the species [22,23]. Recently, a study on the biodegradation of organonitriles reported that benzonitrile can be degraded in benzoic acid and ammonia by nitrilase in microbial systems [24]. Nitrilases were considered absent in algae; however, Lauritano et al. [25] identified for the first time a putative nitrilase in the green microalgae *Tetraselmis suecica* under nutrient-starvation conditions. Moreover, a recent study identified benzoic acid as a new phytohormone improving the growth of *Chlorella regularis* [26]. Thus, a possible enzymatic degradation of benzonitrile was not excluded in our study and the produced ammonia may be assimilated by microalgae [27]. 1,2,3-propanetriol, diacetate is a diglyceride commonly known as diacetin used as a food additive and as a valuable additive to diesel fuel when mixed with other acetins [28]. It is known that soil microorganisms induce lipase–esterase activity for the biodegradation of carboxyl esters [29]. Moreover, some microalgal lipases have been isolated for industrial applications [30] and the transcription of many lipases was induced under abiotic stress (e.g., nutrient starvation) in *Chlamydomonas* [31]. Thus, the complete removal of 1,2,3-propanetriol, diacetate in our system may be through the action of induced microalgal lipases producing glycerol, which in turn may stimulate microalgal growth [32] and assist the degradation of other organic pollutants in CB wastewater such as hydrocarbons [33]. Similarly, Si-based compounds can contribute to the alleviation of numerous environmental constraints in plants by inducing or reinforcing the regulation of secondary metabolites [34,35] and their effective activities are dependent on their chemical and physical characteristics [36,37]. Interestingly, Jeffryes et al. [38] developed a system in which the controlled delivery of Si to the culture of diatom *Cyclotella* spp. enhanced lipid and biomass production. Similar to diatoms, the growth of *Cladophora glomerata* was induced by Si as a required component of the cell walls as in other algae such as *Pediastrum* and *Scenedesmus* spp. [39]. Recently, Van Hoecke et al. [40] demonstrated that Si-based nanoparticles were adhered to the outer cell surface of microalga *Pseudokirchneriella subcapitata* without evidence of particle uptake, concluding that the Si toxicity at high concentration might occur through surface interaction. Hence, it is possible that organosilane compounds in CB wastewater were adsorbed to the microalgal cell wall with some limitations depending on the concentration, chemical group and microalgal strain.

The removal efficiency of CB pollutants named as “others” (UWW abundance: 5.2%) varied among the microalgal strains and these compounds included hydrocarbons and additives such as plasticizers. It has been demonstrated that the microalgae *Scenedesmus obliquus, Chlorella vulgaris* and *Chlamydomonas reinhardtii* could degrade hydrocarbons and the removal capacity varied with the concentration and chemical characteristic of hydrocarbons [41,42,43]. Another study found that photosynthetic pigments in the terrestrial alga *Prasiola crispa* decreased with increasing fuel concentration due to the hydrocarbon lipophilic affinity to the cellular membrane causing chloroplast and/or thylakoid membrane disruption [44]. Concerning plasticizers (e.g., phthalate esters) and their effect on microalgae, Duan et al. [45] demonstrated that environmentally relevant concentrations of dibutyl phthalate stimulated the growth and lipid accumulation in *Chlorella vulgaris*, while higher concentrations damaged cell membranes. Interestingly, another strain of the same species showed a decrease in Chl*a*, growth inhibition and changes in the biosynthesis of relevant proteins at low concentrations [46]. Similarly, the photosynthetic pigments of *Scenedesmus* spp. were reduced under the exposure of dibutyl phthalate at environmentally relevant concentrations affecting microalgal growth and photosynthetic process, while at higher concentrations extracellular soluble proteins were induced acting as osmoregulatory substances [47]. Moreover, the toxicity of plasticizers also depends on their chemical characteristics. For instance, dibutyl phthalate was more toxic than diethyl phthalate in three marine microalgae based on algal growth and Chl*a* content, and the biodegradation was inhibited when these pollutants were mixed [48]. Intriguingly, in our study, all microalgal strains could better remove hydrocarbons and additives at the highest concentration of CB wastewater, highlighting their potential application to remediate oil disasters and toxic plastic-bonded polluted sites. However, more studies are needed to understand how these microalgae degrade or exclude these pollutants from their cells after the uptake, and what kind of defense mechanisms are induced at high CB wastewater concentration.

Nicotine [pyridine, 3-(1-methyl-2-pyrrolidinyl)] is the main tobacco alkaloid and, as expected, it was the most abundant (49.4%) pollutant in CB wastewater. Nicotyrine [pyridine, 3-(1-methyl-1H-pyrrol-2-yl)] is one of the minor alkaloids in tobacco; it can be produced when tobacco is pyrolized [49] and some bacteria can metabolize nicotine into nicotyrine [50]. Both alkaloids represented 52.1% of the total pollutants in CB wastewater and they were generally difficult to remove by microalgae. A recent review highlighted that since 2006, a total of 36 investigations have been performed studying the impacts of CB on aquatic and terrestrial life and lethal impacts seem to be most pronounced in aquatic systems [2]. For instance, leachates from smoked CB over 5 years of decomposition inhibited the growth of the freshwater microalga *Raphidocelis subcapitata* in a bimodal mode, where this inhibition was related to high nicotine concentration at early CB decomposition stage (~30 days postsmoking) and to microplastic release at late stage (5 years) as nicotine concentration declined [51]. Another study using the same species showed that microalgal growth was induced with smoked CB leachates in a concentration-dependent manner from 10% to 75% CB, while at 100% CB (corresponding to 20 butts L^−1^) the growth was inhibited but still higher than control conditions [52]. Studies with marine microorganims showed that CB leachates inhibited the growth of microalga *Dunaliella tertiolecta* in a concentration-dependent manner [52], as well as the Chl concentration of microphytobenthos even at marginal CB concentration (1 butt L^−1^) due to the toxic compounds accumulated in the butt after smoking and the release of microplastics [53]. In our study, CB wastewater concentrations ranged from 1 to 25% (corresponding to 5 to 125 butts L^−1^) and MFA showed that the reduction in Chl*a*, Chl*b* and Chl_total_ in the microalgal strains increased with the low ability to remove nicotine, suggesting that this alkaloid may have the most detrimental effects on these pigments. In fact, chlorophyll biosynthesis in microalgae was inhibited depending on the concentration of nicotine [54,55,56]. In photosynthetic organisms, such as the studied microalgal strains, the light-harvesting pigments (Chl*a* and Chl*b*) effectively capture and transport light energy to the photosynthetic reaction center, while Car absorb the excess of energy protecting the chloroplast from Chl-sensitized photooxidation [57]. Thus, any changes in these pigments can result in energy deficiency to support the growth of microalgae. Similar to Chl, the results of MFA also showed that Car were inhibited in microalgal strains with low ability to remove nicotine. Concordantly, previous studies demonstrated the inhibitory effects of nicotine on Car content, particularly affecting the cyclization of lycopene depending on the nicotine concentration [54,55,58]. Besides nicotine, nicotyrine was also detected in the CB leachates causing the deactivation of nicotine catabolic enzymes in soil microbes [59]. Thus, it is likely that nicotyrine may prevent nicotine catabolism in microalgae and this effect may be pronounced with increasing CB concentration.

Overall, this study highlighted the importance of microalgal strain selection for wastewater remediation, and showed that the strains isolated from similar polluted conditions may necessarily have the best performance, as occurred with LG1, which could not remove efficiently CB-contained alkaloids, and its physiological traits were affected at ≥5% CB similar to the nicotine-resistant mutant of *Chlorella emersonii* [56]. Moreover, microalgal resistance and remediation capacity also depended on the chemical characteristics of pollutants. Here, nicotine was the most resistant pollutant to removal by the microalgae tested and its low removal correlated with the inhibition of photosynthetic pigments affecting microalgal growth. Concerning the optimal conditions for an effective removal of contaminants, our results supported the high performance of *Chlamydomonas* strain F2 to remove organic pollutants at 5% CB wastewater (corresponding to 25 butts L^−1^ or 5 g CB L^−1^) removing 69% of pollutants and maintaining its growth (based on Chl*a*) and pigments at control levels. Further studies are needed to understand the mechanism pathways involved in the removal of pollutants, especially alkaloids.

## 5. Conclusions

A novel solution to recycle filters of cigarette butts (CB) into soilless substrate has previously been proposed, where the CB cleaning process resulted in a contaminated wastewater [7]. In this study, the removal of organic pollutants in CB wastewater by microalgal-based remediation techniques was assessed for the first time, and the data provided a promising approach for wastewater bioremediation, revealing the potential value of the tested microalgal strains for further studies on a larger scale.

## Figures and Tables

**Table 1:** List of microalgal strains.

Strain	Isolation Source	Taxonomic Affiliation	Accession Number	Reference
F1	“Le Morette”, Fucecchio Marshland	*Scenedesmaceae*	OM311002 and OM310999	[11]
F2	“Le Morette”, Fucecchio Marshland	*Chlamydomonas debaryana*	OM311003	[11]
F3	“Le Morette”, Fucecchio Marshland	*Chlamydomonas debaryana*	OM311004	[11]
F4	“Le Morette”, Fucecchio Marshland	*Chlorella sorokiniana*	OM311005 and OM311000	[11]
R1	Private terrace in Pisa, water sample	*Chlorella sorokiniana*	OM311006	[11]
LG1	Recycle cigarette butts substrate	*Chlorella sorokiniana*	ON065975	This work

## Data Availability

Data are contained within the article.
